# Ultrasound assessment of acute Achilles tendon rupture and measurement of the tendon gap

**DOI:** 10.1002/ajum.12384

**Published:** 2024-04-17

**Authors:** Michelle Fenech, Aiyapa Ajjikuttira, Heath Edwards

**Affiliations:** ^1^ School of Health, Medical and Applied Sciences, College of Clinical Sciences, Central Queensland University Brisbane Campus, 160 Ann Street Brisbane 4000 Queensland Australia; ^2^ Department of Medical Imaging Royal Brisbane and Women's Hospital Herston, Brisbane 4006 Queensland Australia

**Keywords:** Achilles tendon rupture, Achilles tendon tears, Kager's fat pad, musculoskeletal ultrasound, plantaris tendon, tendon gap measurement

## Abstract

Achilles tendon rupture is a common sports‐related injury which can carry significant morbidity to patients. Ultrasound remains the workhorse of imaging as it can confirm and localise the extent of Achilles tendon injury. The sonographic anatomy, both normal and ruptured sonographic appearances, as well as sonographic technique must be appreciated to accurately image and report findings, critical to patient management. Particular attention should be applied to the measurement of the diastasis between acutely ruptured tendon ends as this information can assist with informing the decision of conservative vs. operative management. Further work is necessary to standardise the measurement technique including correlating the degree of plantarflexion of the foot with the sonographic tendon gap measures.

## Introduction

Rupture of the Achilles tendon is one of the most common sports‐related injuries with an annual incidence of 5–50 events per 100,000 persons.^1^, ^2^, ^3^ Achilles tendon rupture is debilitating because of its involvement in ambulation and activities in athletes and non‐athletes alike.^2^ Ultrasound imaging plays an important role in confirming, locating and defining the extent of an acutely ruptured Achilles tendon.^4^, ^5^, ^6^ The operative or non‐operative management of acute Achilles tendon ruptures is a much‐debated topic.^7^, ^8^, ^9^, ^10^, ^11^ Two major indications for surgical treatment are tendon gap formation of more than 10 mm when the foot is in plantar flexion, and treatment initiation more than 24 hours following an injury.^11^

Surgical repair of the ruptured Achilles tendon has not been demonstrated to be associated with better outcomes than non‐operative treatment at 12 months.^12^ The main reason for supporting operative treatment is lower re‐rupture rates.^13^ However, recent meta‐analyses have shown that early functional rehabilitation protocols reduce the re‐rupture rate in non‐operatively treated patients to a similar level in patients treated by surgery.^14^, ^15^ Operative treatment, however, results in higher risk of complications such as infection, iatrogenic sural nerve injury, poor wound healing, scar tissue formation and adhesions.^16^

Sonographic demonstration of dynamic movement of acutely ruptured Achilles tendon ends with altering degrees of plantar flexion aids in confirming rupture diagnosis.^17^ However, there is a paucity of studies investigating the dynamic sonographic visualisation of acutely ruptured Achilles tendon ends and measurement of the tendon gap.^18^ Increasingly, sonographic measurements of the gap size between ruptured Achilles tendon ends are being used to guide patient management.^19^, ^20^ Therefore, a structured sonographic approach to measuring the gap between tendon ends under varying degrees of plantarflexion is required to ensure consistent reporting of sonographic findings to appropriately inform the decision of conservative or operative treatment pathways.^21^ This paper outlines the sonographic anatomy, imaging technique and imaging findings required that should be appreciated to assess an acutely injured Achilles tendon, including how to measure the gap between ruptured Achilles tendon ends.

## Achilles tendon structural anatomy

The Achilles tendon is the longest, thickest and strongest tendon in the body due to its collagen content and cross‐sectional area.^22^ Despite its tensile strength, it is affected by spontaneous rupture.^23^ The Achilles tendon is formed by the fusion of the aponeurosis of the two heads of the gastrocnemius with the tendon of the deeper soleus.^4^ The free tendon subsequently consists of three subtendons arising from the three muscular heads of the triceps surae.^24^, ^25^ During its course, the three Achilles subtendons twist and rotate approximately 90° internally as they pass from proximal to distal.^24^ This twisting occurs in a counterclockwise fashion for the right Achilles tendon and clockwise fashion for the left.^26^, ^27^ The gastrocnemius fibres consequently insert laterally, towards the posterior calcaneus, and the soleus fibres inserting anteriorly at the medial aspect of the calcaneus.^25^, ^26^, ^28^ The calcaneal insertion is a true enthesis with fibrocartilage intermeshing with the marrow of the calcaneus.^28^

The Achilles tendon is divided into proximal myotendinous junctions (MTJ), the mid portion of the tendon called the ‘free tendon’ and its distal insertion and enthesis onto the calcaneus. The Achilles tendon has a relatively hypovascular (watershed) zone in its mid‐section of the free tendon, approximately 2–6 cm proximal to its distal insertion.^19^ This watershed area of the free tendon is theorised to reduce the speed of micro‐trauma repair, lead to degeneration of the tendon, and contribute to, or predispose it to rupture.^29^ This theory, however, needs further investigation.^30^ Achilles tendon perfusion can be compromised during tendon stretching and contraction.^4^ With increasing age, there is decreased collagen‐crosslinking and weakening of the tensile strength of the Achilles tendon which can pre‐dispose it to rupture.

**Figure 1:**
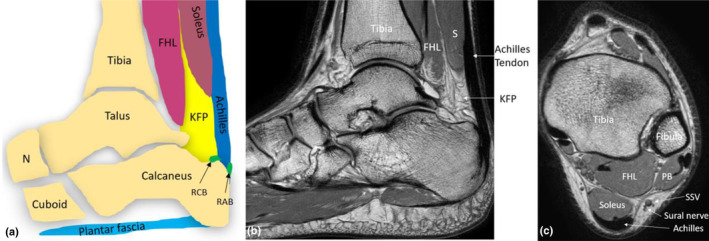
Anatomical schematic (a) and sagittal proton density (PD)‐weighted magnetic resonance (MR) image (b) demonstrating the long axis of the Achilles tendon. (c) Axial PD MR image of the Achilles tendon. FHL, flexor hallucis longus muscle; KFP, Kager's fat pad; PB, Peroneus brevis muscle; RAB, Retro‐Achilles bursa; RCB, retro‐calcaneal bursa; S, soleus muscle; SSV, small saphenous vein.

The Achilles tendon sits superficial to the pre‐Achilles fat pad, also known as Kager's fat pad (KFP), as it occupies the space of Kager's triangle. KFP is located anteroinferior to the distal soleus muscle, and posterior (superficial) to the distal flexor hallucis longus (FHL) muscle and tendon, the posterior ankle joint, talus and calcaneus.^4^ KFP has the role of lubricating motion between the Achilles tendon and calcaneus and is an important sonographic landmark for imaging the Achilles tendon.^31^ Deep, or anterior to the distal Achilles tendon, just proximal to its insertion, lies the synovial lined retrocalcaneal bursa (RCB), also known as the pre‐Achilles bursa. The inferior portion of Kager's fat pad moves inferiorly into the region of the RCB during plantarflexion.^32^ Steroid injections can be used for the treatment of severe retrocalcaneal bursitis; however, corticosteroids have been implicated in delaying healing of the Achilles tendon.^33^ Corticosteroids have been proposed to be associated with an increased risk of Achilles tendon rupture, particularly when concomitantly used with quinolone antibiotics, but this theory needs to be further investigated.^34^, ^35^, ^36^ The retro‐Achilles bursa (RAB), also called the superficial calcaneal bursa, is located superficial to the distal Achilles tendon insertion and deep to the skin (Figure 1).

**Figure 2:**
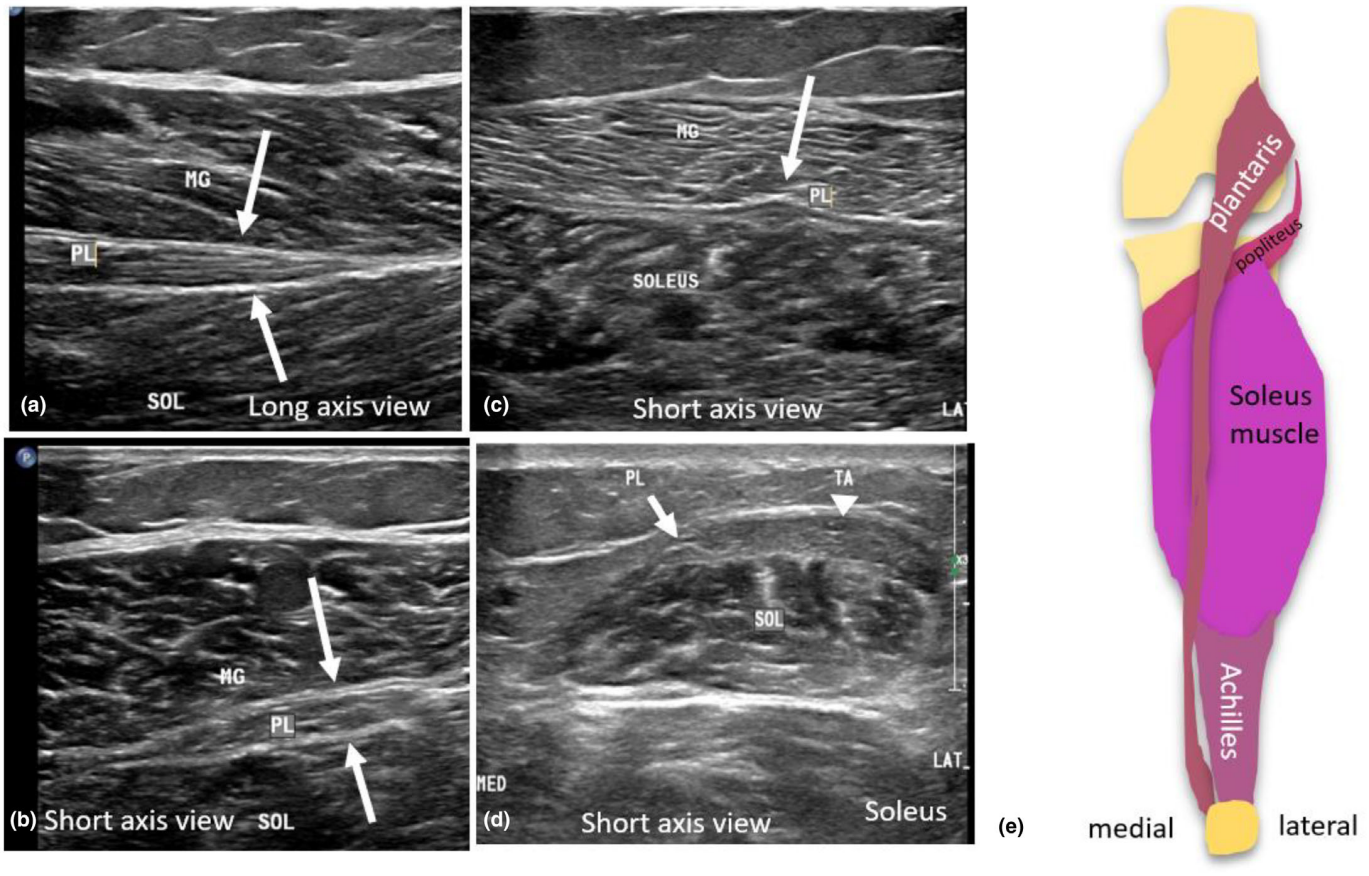
Sonographic imaging of the plantaris muscle and tendon. (a) Long‐axis sonographic image of the plantaris (PL) muscle (white arrows) in the proximal calf between the medial gastrocnemius muscle (MG) and soleus (SOL). (b) Short‐axis image of the plantaris muscle (white arrows). (c) Short‐axis image of the plantaris tendon (PL) outlined by white arrows between the medial gastrocnemius and soleus muscles. (d) Short‐axis image of the plantaris tendon (PL) located along the medial aspect of the Achilles tendon (TA – indicated by the white arrowhead), forming superficial to the soleus muscle. (e) anatomical schematic of the plantaris muscle and tendon. MED, medial aspect of short‐axis image; LAT, lateral aspect of short‐axis image.

The plantaris muscle and tendon are variably reported to be present in up to 90% of individuals.^37^ Arising from the lateral supracondylar ridge of the femur, the plantaris has a small muscle belly that passes obliquely between the gastrocnemius and soleus muscles. The distal tendon of the plantaris is small but long, extending distally between the hypovascular area of the soleus muscle, while overlying the medial gastrocnemius muscle close to the medial aspect of the Achilles tendon.^37^ The plantaris tendon has a variable insertion onto either the posteromedial aspect of the calcaneus (anteromedial to the Achilles tendon), the Achilles tendon itself, the flexor retinaculum or the medial paratenon.^4^ Due to its shape and small cross‐sectional area, it can be mistaken for a nerve and thus sometimes referred to as the ‘fool's nerve’.^37^ The plantaris tendon often remains intact when the Achilles tendon is ruptured, and its intact fibres should not be mistaken for remnant Achilles tendon fibres.^38^ Collectively, the Achilles–plantaris complex can be referred to as the triceps surae complex, which acts to flex the foot (Figure 2).

The crural fascia (fascia cruris) envelops muscles of the lower leg forming the anterior, posterior and lateral muscular compartments. In the posterior leg, the crural fascia splits into superficial and deep laminae, generating a large fascial sleeve which encloses both the Achilles tendon and Kager's fat pad.^39^ The free Achilles tendon lacks a true tendon sheath and is rather, enveloped by a richly vascularised and innervated paratenon.^40^ The paratenon can be appreciated as “U” shaped, deep to the crural fascia and capping the posterior (superficial), medial and lateral aspects of the Achilles tendon, sparing its anterior (deep) surface.^4^, ^39^ Vascularised by branches from the posterior tibial and peroneal arteries, the paratenon is theorised to provide a pathway of vascular supply to the Achilles tendon which assists with healing when the tendon is injured.^41^, ^42^ The paratenon and crural fascia can become torn and/or inflamed in association with acute Achilles tendon ruptures.^40^ Further studies are required to clarify the detailed distinction between the crural fascia and the paratenon, and its relationship to the plantaris tendon.

**Figure 3:**
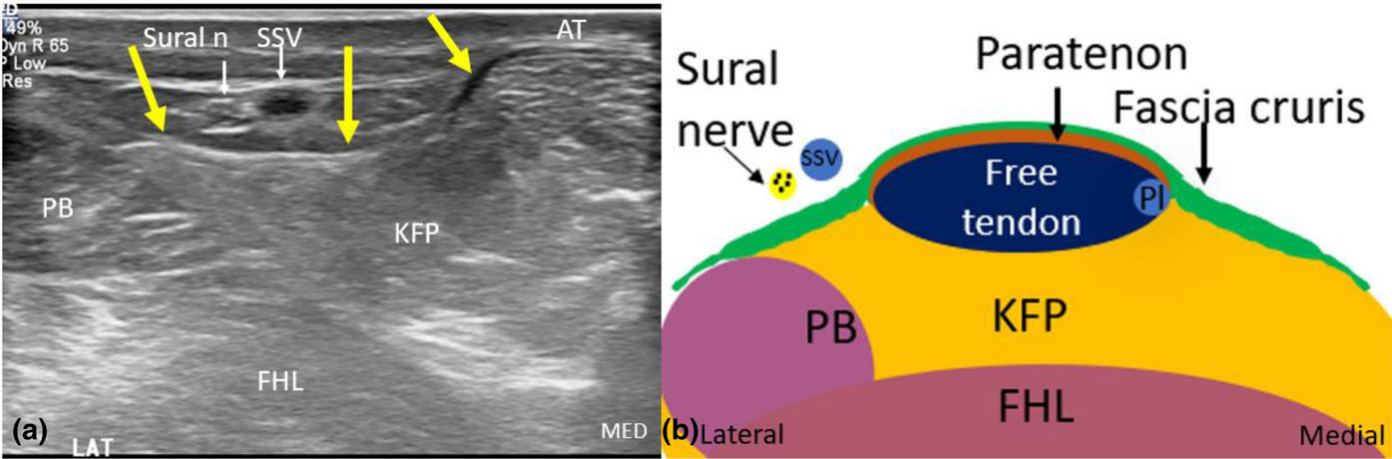
Short‐axis sonographic image (a) and anatomical schematic (b) of the Achilles tendon, fascia cruris, sural nerve and paratenon at the level of the free Achilles tendon. The sonographic image is acquired just to the lateral aspect of the Achilles tendon. This allows the fascia cruris to be identified extending superficial to the free Achilles tendon and paratenon and extending laterally (outlined by yellow arrows) to the overlie the peroneus brevis muscles, and deep to the sural nerve (n) and small saphenous vein (SSV). AT, Achilles tendon; FHL, flexor hallucis longus muscle; KFP, Kager's fat pad; LAT, lateral aspect of image; MED, medial aspect of image; PB, peroneus brevis muscle; Pl, plantaris.

An important neurological structure that warrants attention in cases of Achilles tendon injury is the sural nerve. The sural nerve is sensory and innervates the posterolateral distal calf and lateral foot. In the distal posterior calf, it is superficial in location, coursing near the small saphenous vein (SSV) and lateral to the Achilles tendon, posterior and superficial to the soleus and peroneal muscles. As the sural nerve runs close to the Achilles tendon, it can be affected by Achilles tendon pathology and may be injured during surgical repair.^43^ Additionally, when the Achilles tendon is acutely ruptured, the sural nerve is vulnerable to compression by an adjacent haematoma, particularly when the fascia crura is concurrently torn^44^ (Figure 3).

## Sonographic appearance of the normal Achilles tendon and surrounding structures

Ultrasound imaging is used for the assessment of the Achilles tendon as it is inexpensive, easily accessible, and when used appropriately, as sensitive as magnetic resonance (MR) imaging.^45^ Ultrasound as an imaging modality is, however, operator dependent. Due to the superficial location of the Achilles tendon, a high‐frequency linear array transducer (>12 MHz) should be used to acquire the highest resolution images of the tendon and surrounding structures. Sonographic assessment is performed with the patient prone, and their feet hanging over the end of the examination bed, as this allows plantar and dorsiflexion of the foot to be performed whilst conducting imaging. Comparison to the asymptomatic contralateral side is easy to perform, and uninjured Achilles tendons should appear symmetrical between limbs of the same person. The tendon itself is imaged from the muscles that form it and their MTJ to its distal calcaneal insertion in short‐ and long‐axis planes. Colour and power Doppler should be used to assess the vascularity of the tendon and surrounding structures. Importantly, the Achilles tendon should be imaged perpendicular to the incident sound, to avoid anisotropic artefacts which may mimic pathology and tears. Ultrasound imaging has the advantage over MR imaging as dynamic assessment is easily performed. The width, thickness, length and cross‐sectional area of the Achilles tendon are dependent on the height, weight, tibial length, foot size and activity level of an individual; however, limb dominance does not appear to have an effect on Achilles tendon size.^46^

**Figure 4:**
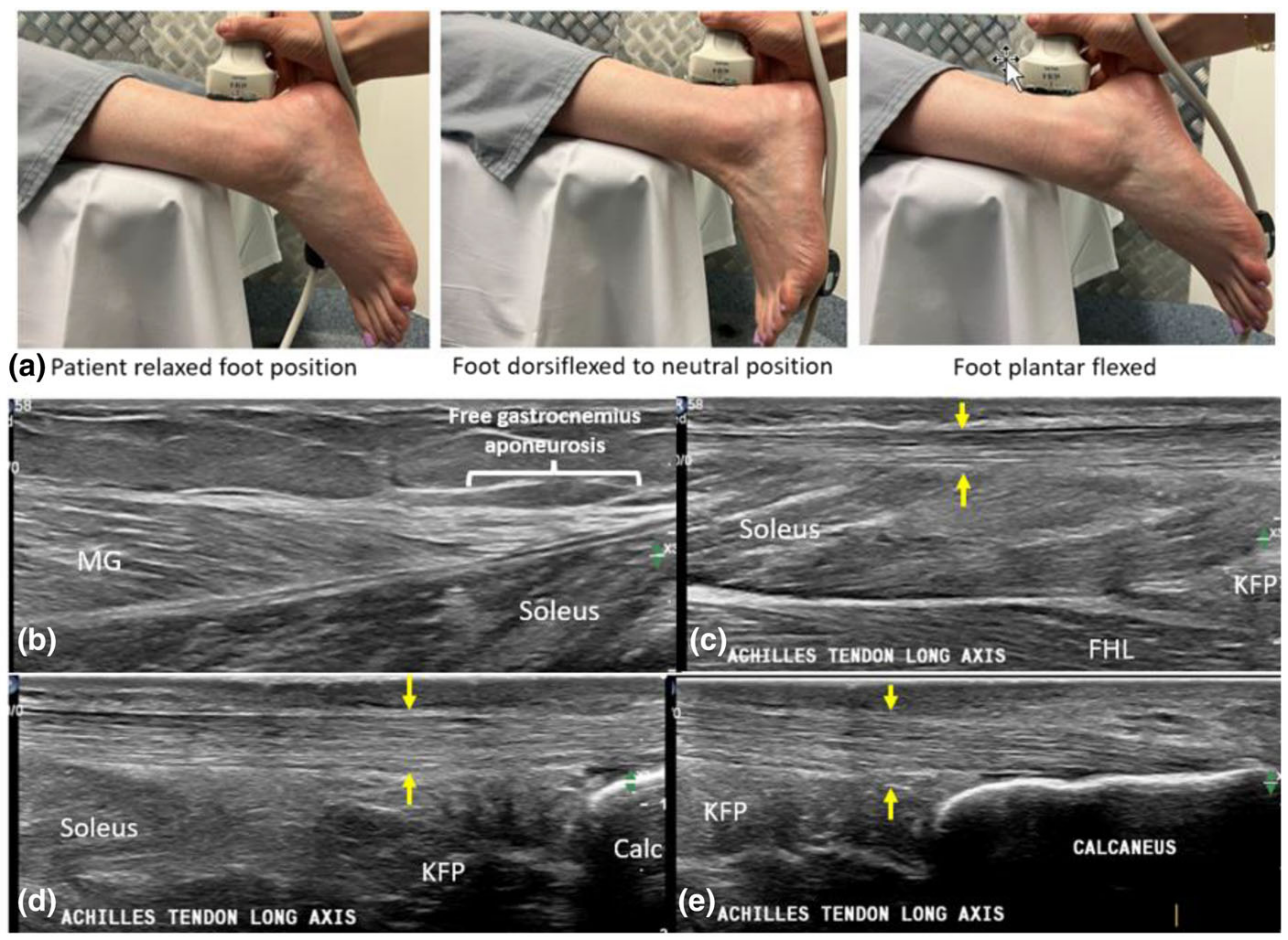
Long‐axis sonographic imaging of the uninjured Achilles tendon. (a) Positioning to allow different degrees of foot plantar and dorsiflexion. (b) Long‐axis sonographic image of the medial gastrocnemius (MG) and free gastrocnemius aponeurosis. (c) Proximal Achilles tendon and myotendinous junction of the Achilles to the soleus. (d) Long‐axis image of the free Achilles tendon (yellow arrows) superficial to Kager's fat pad. (e) Long‐axis image of the distal Achilles tendon and enthesis. Achilles tendon, yellow arrows; Calc, Calcaneus, FHL, Flexor Hallucis Longus muscle; KFP, Kager's fat pad; MG, medial gastrocnemius.

Proximal to distal long‐axis imaging allows assessment of the distal gastrocnemius muscles, their echogenic aponeuroses and echogenic free gastrocnemius–Achilles junctions.^47^ The soleus–Achilles MTJ is then visualised and typically demonstrates a unipennate arrangement of the soleus muscle fibres obliquely joining the superficially located Achilles tendon in long axis.^4^ The soleus muscle thickness reduces as imaging is extended distally. The free Achilles tendon, distal to the soleus MTJ and superficial to KFP, in its normal state sonographically appears to have relatively parallel borders, and a homogenous, organised fibrillar echotexture with parallel alignment of the collagen fibres (echogenic bright bands) alternating with hypoechoic bands of extracellular matrix.^4^ In cases when an accessory soleus muscle is present, sonographic distortion or change in shape to the Kager's fat pad may be seen. The paratenon and crural fascia overlie the superficial, medial and lateral borders of the free Achilles tendon, and in their normal state, can be difficult to sonographically discriminate. Dynamic evaluation of the tendon and surrounding structures in long axis is achieved by actively or passively plantar‐ and dorsiflexing the foot. The uninjured Achilles insertion onto the calcaneus at its enthesis is about 10 mm long^4^ (Figure 4).

In normal states, the RCB when imaged in long axis is comma‐shaped, with the tail of the comma extending between the Achilles and calcaneus. The RCB can be imperceptible, or contain up to 3‐mm fluid measured from superficial to deep (or posterior to anterior) in asymptomatic patients, which can change shape with plantar and dorsiflexion.^4^ RCB distension greater than 3 mm can occur due to inflammation or haemorrhagic bursitis and can mimic Achilles tendon pathology clinically.^5^

**Figure 5:**
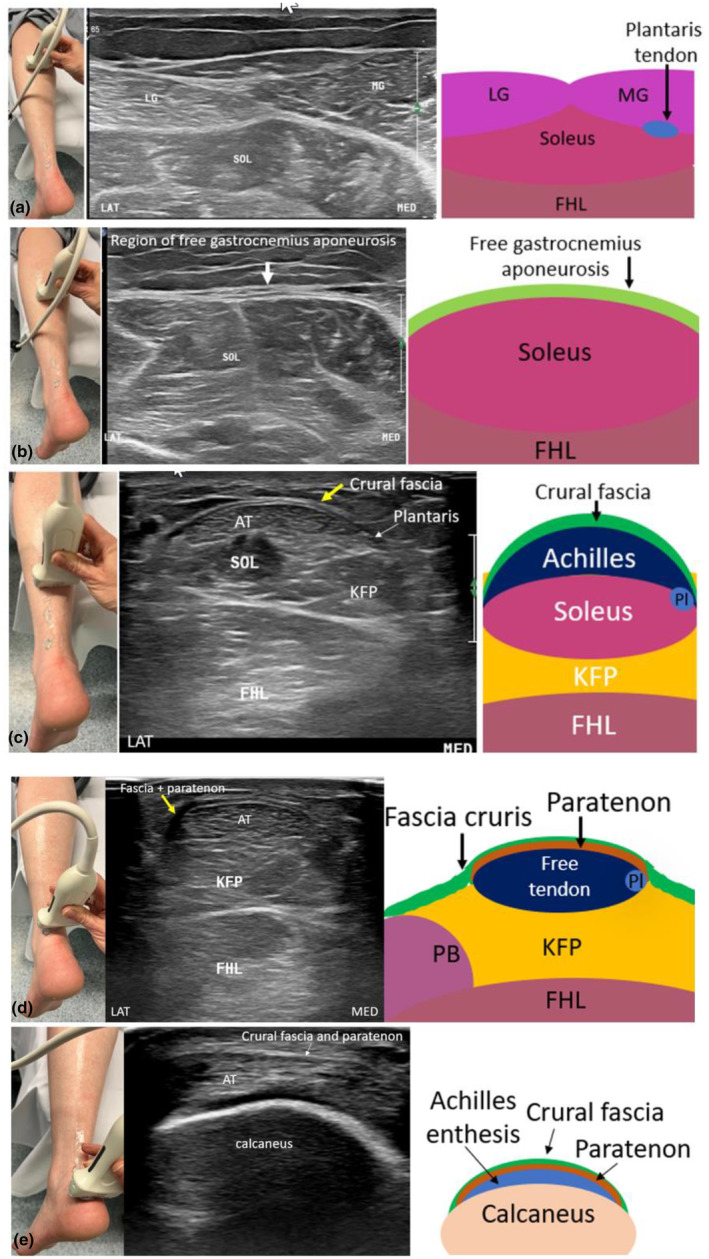
Proximal to distal short‐axis sonographic images with accompanying anatomical schematics at the same level, of healthy, uninjured triceps surae muscles and Achilles tendon. (a) At the triceps surae muscle level, the lateral gastrocnemius, medial gastrocnemius and soleus muscles are observed. (b) The free gastrocnemius aponeurosis is observed extending 10 mm distal to the gastrocnemius muscles, superficial to the soleus muscle. (c) The soleus–Achilles myotendinous junction which attaches to the deep aspect of the Achilles tendon and the deeper flexor hallucis longus muscle can be identified. (d) The mid‐portion of the Achilles tendon and free Achilles tendon is located superficial to Kager's fat pad. The paratenon and located superficial to the Achilles tendon. (e) The distal Achilles tendon inserts onto the calcaneus via the enthesis. AT (dotted arrow), Achilles tendon; FHL, flexor hallucis longus; KFP, Kager's fat pad; LAT, lateral aspect of image; LG, lateral gastrocnemius; MG, medial gastrocnemius; MED, medial aspect of image; Pl, plantaris tendon; SOL, soleus muscle.

Short‐axis sonographic imaging allows the medial and lateral portions of the Achilles tendon and surrounding and associated structures to be appreciated. Sonographically, the tendon component appearances and shape can be seen to alter when imaging from proximal to distal in short axis. Proximally, the tendon is identified as a flattened band superficial to the soleus. At the free tendon level, superficial to Kager's fat pad, the Achilles demonstrates an oval shape in short axis. Distally, at its insertion onto the posterior surface of the calcaneus, the Achilles tendon becomes convex shaped. Close to the medial aspect of the Achilles tendon, the plantaris tendon may be identified in short‐axis sonographically as an oval structure. The plantaris tendon is best identified initially proximally, where it is located between the medial gastrocnemius and soleus muscles, and then followed distally along the medial aspect of the Achilles tendon. The sural nerve is identified in short axis, lateral to the region of the Achilles tendon, superficial to the crural fascia and should demonstrate a fascicular, honeycomb sonographic appearance. The hypoechoic fascicles of the sural nerve should appear similar in size surrounded by echogenic perineurium and epineurium^44^ (Figure 5, Video S1).

## Acute Achilles tendon rupture

Acute Achilles tendon tears are not uncommon in young, active people and may be categorised as a spectrum consisting of microtears, partial and interstitial tears and complete ruptures.^21^, ^28^ Achilles tendon ruptures are generally defined to occur at three locations: (i) proximally at the MTJ, (ii) At the level of the ‘free tendon’ and (iii) distally at the calcaneal insertion/enthesis.^5^ The most commonly reported site of rupture is at the mid‐tendon, approximately 4–6 cm proximal to its insertion.^48^ However, further studies reporting the prevalence of Achilles tendon rupture sites are required.

A clinical diagnosis of acute Achilles tendon rupture is made by carefully taking a patient history and undertaking a physical examination. Injury usually occurs after forced dorsiflexion of the ankle, such as a take‐off injury.^19^ Patients typically describe a sensation of a sudden ‘snap’ or ‘pop’ in the back of their ankle and acute severe pain, usually localised to the rupture site. Commonly, patients state they thought someone had kicked, shot or even bitten them in the back of their leg, or incorrectly assumed something had been thrown at their leg.^19^, ^49^ An audible ‘pop’ may be heard at the time of injury, followed by falling to the ground due to difficulty weight bearing, and an inability to walk or tiptoe on the injured side post‐injury.^4^

Following an acute rupture, the Achilles tendon is best physically examined with the patient lying prone on a bed, or kneeling on a chair, with their feet hanging over the end of the bed or chair.^19^ A palpable defect in the region where the normally tense, rope‐shaped Achilles tendon should be present is usually visualised or felt. Palpation of the gap between torn Achilles tendon ends can be difficult to detect in obese patients, or those with marked peritendinous oedema that may obliterate the appreciable gap.^7^ Bruising or swelling associated with injury can take up to a week to become visible.

On physical assessment, plantarflexion of the foot is weak or absent when the patient is asked to perform plantar flexion against resistance.^4^ Physical tests to assess the Achilles tendon in the acute setting include the ‘calf squeeze’ test, also called the Simmonds (British) or Thompson (North American) test.^7^ When the calf is squeezed, and plantar flexion of the foot does not occur, when compared to the unaffected leg, the suspicion of a complete tear of the Achilles tendon is raised.^4^ However, if the plantaris tendon is intact, the ‘calf squeeze’ test may result in plantar flexion of the foot, limiting the sensitivity of this test. The Matles, or ‘knee flexion test’, can also be performed. The patient is positioned prone and asked to actively flex their knee to 90°. During this movement, the test is positive when foot of the affected side falls into a neutral or dorsiflexed position compared to the contralateral ankle.^50^ However, a false‐positive test may occur when there is neurologic weakness.^23^

**Figure 6:**
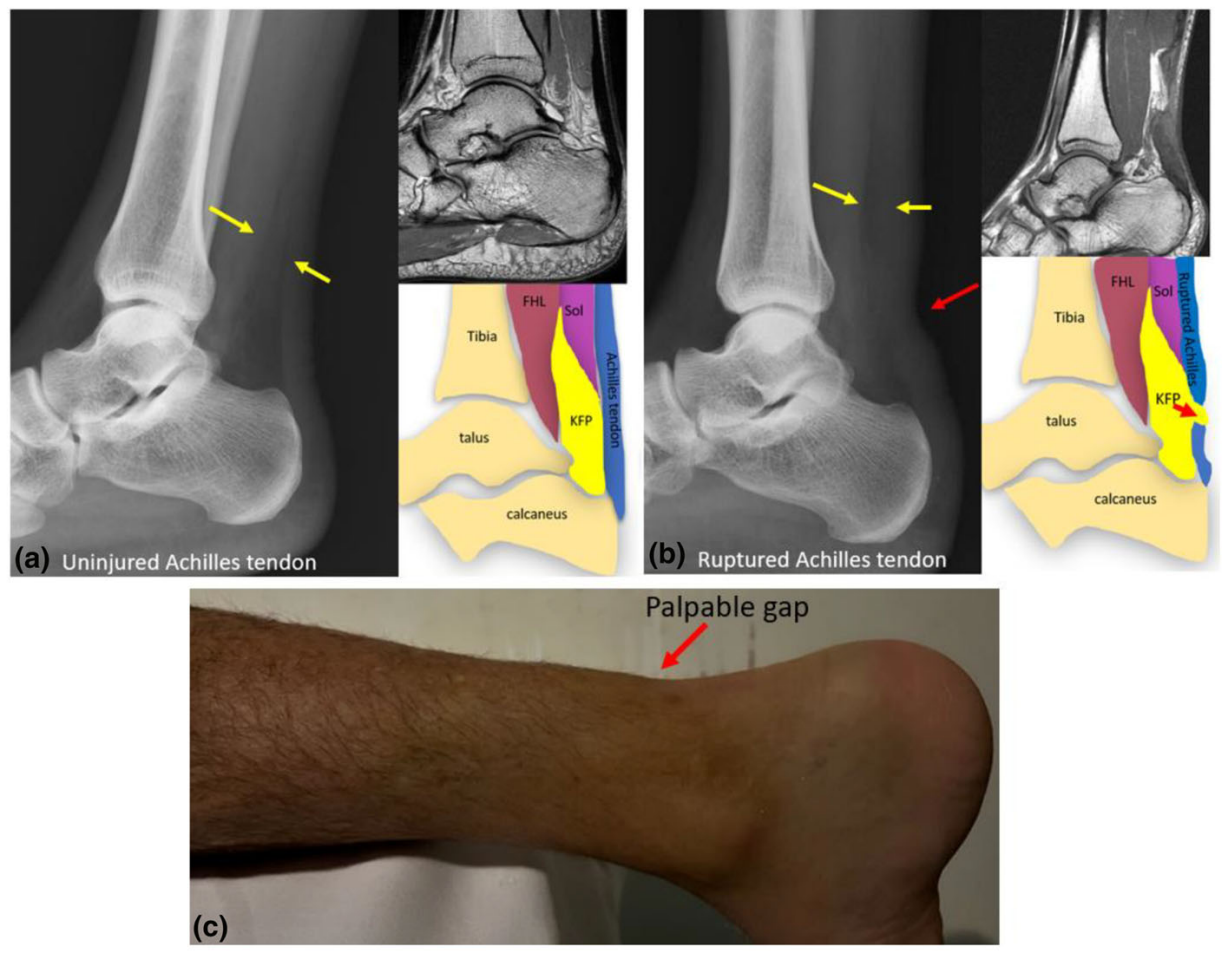
Radiographs and magnetic resonance (MR) images demonstrating an intact and ruptured Achilles tendon. (a) Intact Achilles tendon. A clearly defined Kager's triangle is observed as a clearly defined area of low density (yellow arrows) on a lateral radiograph. On a sagittal MRI, Kager's triangle is clearly defined. (b) When the Achilles tendon is ruptured, the margins of Kager's triangle, outlining the region of the pre‐Achilles fat pad become distorted on a plain lateral radiograph (yellow arrows). On a sagittal MR imaging, Kager's fat pad herniates into the gap between ruptured tendon ends. (c) Photograph of the palpable region of a ruptured Achilles tendon. FHL, flexor hallucis longus muscle; KFP, Kager's fat pad; Sol, soleus muscle.

Diagnostic imaging is useful to confirm a clinically suspicious acute Achilles tendon rupture. Plain radiographs of the ankle can be performed following an acute injury to evaluate the presence of any concomitant bony fracture and assess the integrity of the Achilles tendon. Plain lateral radiographs may reveal an irregular outline or configuration of the low‐density appearing KFP as the borders become distorted when the tendon is ruptured.^23^ MR imaging can be used to demonstrate tendon disruption; however, static MR imaging does not demonstrate dynamic movement of the tendon ends (Figure 6).

### Immobilisation of the Achilles tendon following acute rupture

At the time of initial presentation following an acute injury where Achilles tendon rupture is clinically suspected, patients are placed in a below‐knee ‘equinus’ cast, front‐slab or controlled ankle movement (CAM) boot. This results in the foot resting in a plantarflexed position.^12^ Reference is occasionally made to immobilisation in the ‘gravity equinus’ position, which is the position that the foot naturally adopts when unsupported.^8^ Immobilisation provides stability but also ensures that the Achilles tendon ends are positioned as closely opposed together as possible. This can facilitate healing, and limit tendon elongation.^11^ Tendon elongation can lead to reduced plantar flexion of the ankle (reduced heel raise height) and gait impairment and may result when scar tissue from healing fills a wide gap between ruptured tendon ends.^51^

**Figure 7:**
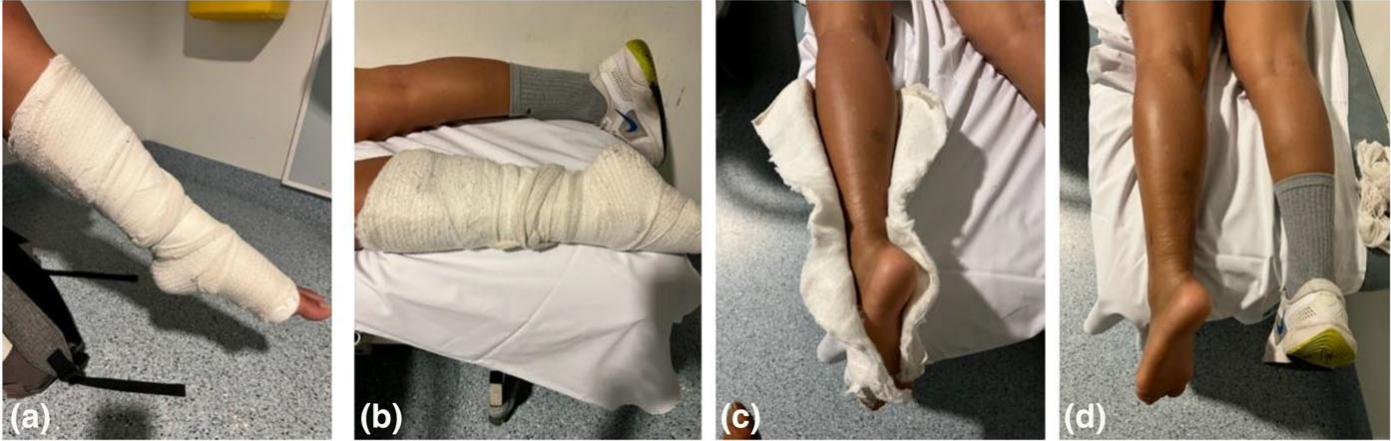
Immobilisation for a clinically suspicious ruptured Achilles tendon. (a) A front slab with the foot in plantar flexion. (b) For sonographic imaging, the patient is positioned prone with their foot hanging over the end of the bed. (c) The front slab is cut at the posterior aspect to allow visual inspection and sonographic imaging. (d) The front slab is removed to allow dorsiflexion and plantar flexion during sonographic imaging.

The optimal angle of plantarflexion for initial immobilisation of suspected Achilles tendon rupture is still yet to be clearly defined. Maximal patient‐tolerated plantar flexion can be utilised, but more commonly the foot is placed in a 30° to 40° plantarflexed position. Full plantar flexion may not be needed to fully oppose ruptured Achilles tendon stumps, and in some cases, may result in an overlap of tendon stumps.^52^ In addition, greater degrees of plantar flexion, such as 45° to 50° can result in increased flexion of the hip and knee to swing the foot, causing alteration in gait mechanics^52^ (Figure 7).

## Ultrasound imaging of suspected acute Achilles tendon ruptures

### Ultrasound discrimination between Achilles tendon ruptures and partial tears

Ultrasound is useful in defining the difference between a complete and partial Achilles tendon tear.^5^ Partial tears are defined as the partial discontinuation of the Achilles tendon.^53^ They may be located at the surface of the tendon or intratendinous.^51^ Partial surface tears tend to be related to tendon degeneration, have a vertical orientation and are more commonly located in the dorsal aspect of the tendon, affecting the subtendon from the medial head of the gastrocnemius.^54^ Sonographically, a partially torn tendon can appear thickened with disruption to the tendon fibrillar echotexture and a gap between fibres. However, part of the tendon will still be observed to be continuous between proximal and distal ends. Intrasubstance (intratendinous) tears are more commonly identified parallel to the long axis of the tendon in the anteromedial tendon, mostly affecting the subtendons from the lateral gastrocnemius and soleus muscles.^51^ Sonographically, intrasubstance tears are often located within thickened tendons, are related to tendinopathic changes and appear as longitudinal hypoechoic gaps or defects between tendon fibres, where the remaining tendon can demonstrate a preserved fibrillar pattern.^4^

## Long‐axis sonographic imaging of ruptured Achilles tendon

**Figure 8:**
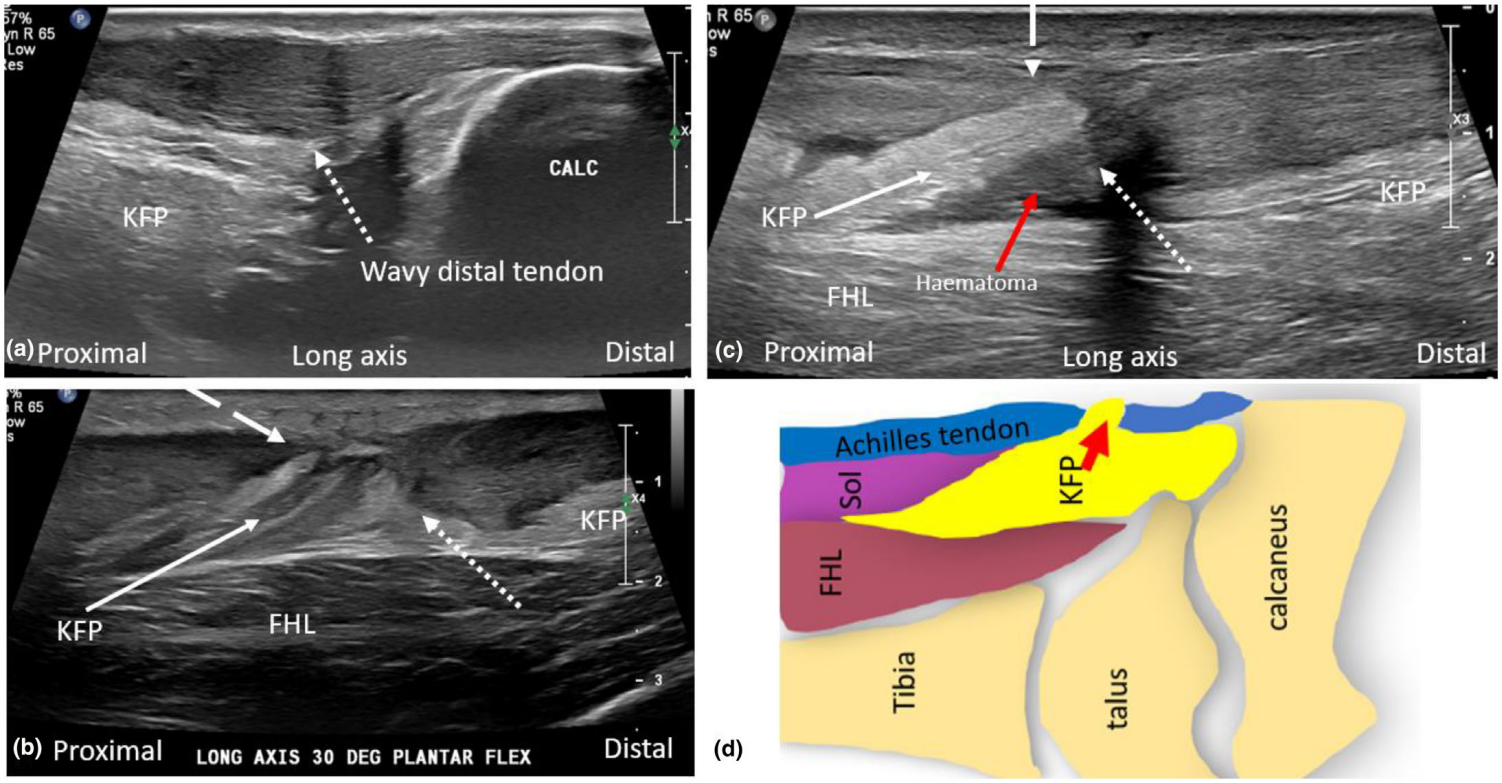
Long‐axis sonographic imaging of acutely ruptured Achilles tendon (mid tendon). (a) The tendon distal to the rupture (dotted arrow) appears wavy and not taut. (b, c) At the tendon rupture site, Kager's fat pad (KFP) can be seen herniating superficially and inferiorly through the gap between the proximal (dashed arrow) and distal (dotted arrow) tendon ends. (d) Depiction of the herniation of the KFP in a superficial and downward direction between ruptured Achilles tendon (blue) ends. Calc, calcaneus; FHL, Flexor hallucis longus muscle; KFP, Kager's fat pad; Sol, soleus muscle.

When an acute rupture of the Achilles tendon is present, the proximal and distal ends of the tendon will not appear continuous with long‐axis sonographic imaging. Additionally, the fibrillar tendon echo pattern will be disrupted, and a focal defect extending between the superficial and deep borders of the tendon and between proximal and distal torn tendon edges will be observed.^4^ The paratenon, crural fascia and plantaris tendon can be concurrently torn when the Achilles tendon is ruptured and may appear discontinuous.^40^ If the paratenon and crural fascia remains intact, it may become elevated from the underlying torn tendon due to haematoma formation.^55^ As the Achilles tendon ruptures, vessels also rupture and a haematoma may form.^56^ The echogenicity of an associated haematoma can be variable and also alter over time (Figure 8).

When sonographically examining a patient with a suspected Achilles tendon rupture, as the patient is positioned prone, it is important to ensure the correct labelling of right and left legs. Ultrasound imaging can be challenging immediately following an acute rupture due to the swelling, pain, tenderness and haematoma surrounding the tear which can fill the space between tendon stumps and cause scattering of sound waves, degrading image quality. Long‐axis sonographic imaging is utilised from the distal enthesis to the proximal MTJ of the soleus and gastrocnemius muscles to confirm, identify and localise the rupture site relative to the insertion onto calcaneus. A distal rupture within 2 cm of the enthesis in a younger population is less common and more challenging than midportion ruptures to treat, due to minimal amounts of distal soft tissues and the likelihood of pre‐existing tendinopathy.^51^, ^57^ The torn and frayed proximal and distal tendon ends relative to the rupture site, may appear wavy, buckled and not taut. Longitudinal splits may be visualised in the proximal and distal ruptured tendon ends. An intact plantaris tendon should not be mistaken for intact residual Achilles tendon fibres and misinterpreted as an incomplete tear. In long‐axis imaging, the plantaris tendon can be demonstrated by fanning ultrasound imaging from lateral to medial.

In the acute phase post‐Achilles tendon rupture, the intervening gap between torn tendon and paratenon ends may not be sonographically obvious as a haematoma filling the gap may appear isoechoic. KFP can angle distally towards or herniate into the ruptured tendon defect from the deep to superficial aspect. The KFP becomes inflamed and usually appears echogenic and heterogenous and can be a good sonographic landmark when identifying a tendon rupture site. Areas of posterior acoustic shadowing at the torn tendon ends may occur, and aid in highlighting the rupture site. Over time following the injury, the frayed tendon stumps can become more regular and the separation between tendon ends can become more apparent.^51^ The tendon ends may not align in the horizontal plane. One stump, usually the more proximal, may be observed to be angled superficially and the other, usually the more distal stump, angled deep (Video S2). This can result in anisotropy of the tendon ends, so varied transducer angulation, may be required to correctly identify the true tendon stump. Retraction of tendon ends at the rupture site may be observed and can depend on the degree of dorsiflexion of the foot. With extreme plantar flexion in some patients, overlapping of tendon ends may be visualised.^28^

## Short‐axis imaging of acutely ruptured Achilles tendon

Short‐axis sonographic imaging of a ruptured Achilles tendon acquired from proximal to distal can demonstrate the tendon rupture site as well as any concurrent injuries such as muscle, MTJ and plantaris tendon tears, paratenon of crural fascia tears or sural nerve irritation. A cine clip of imaging from proximal to distal helps demonstrate different levels at which concurrent injuries may occur (Video S3).

**Figure 9:**
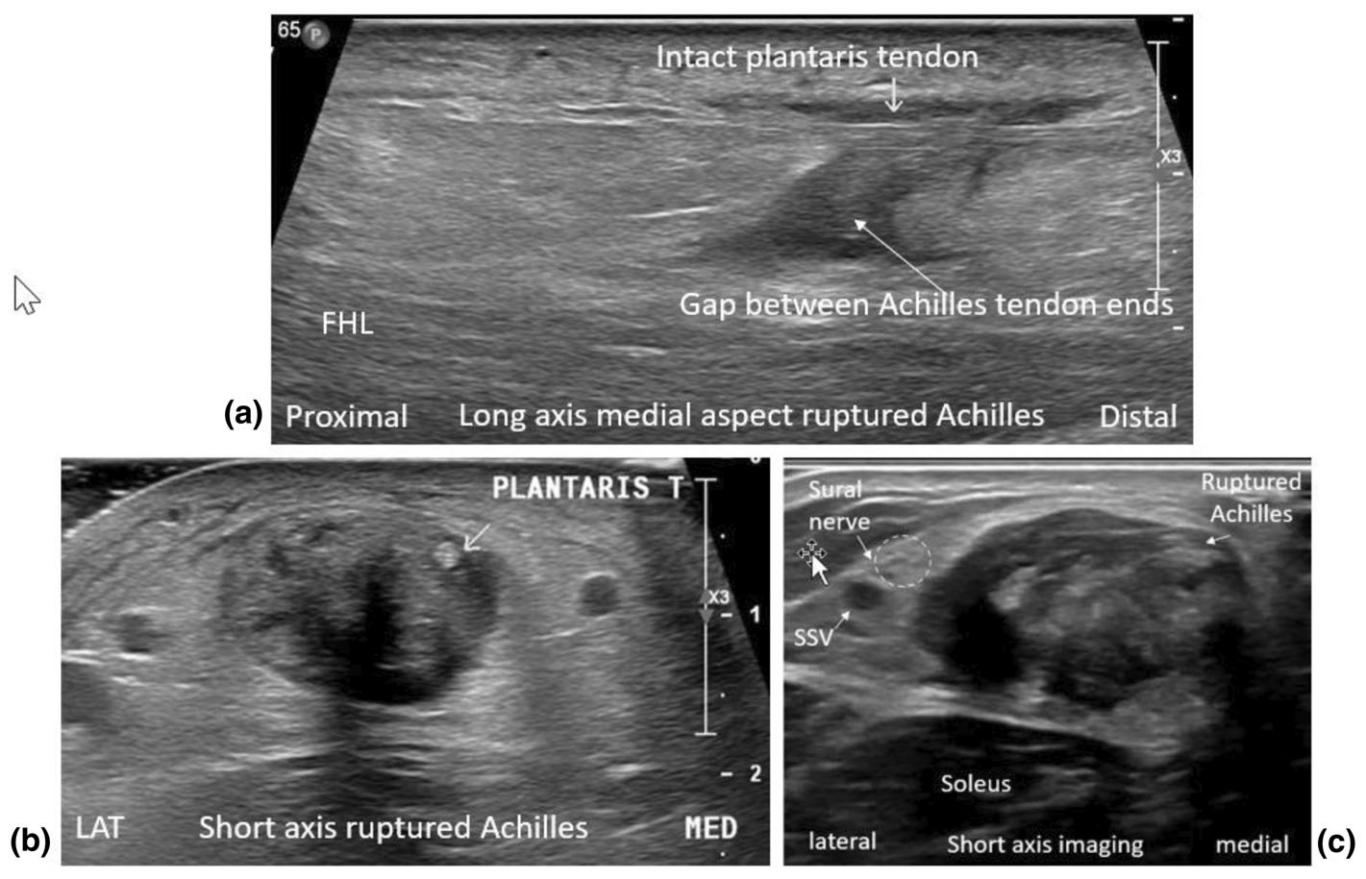
Long‐ and short‐axis imaging of ruptured Achilles tendons and associated findings. (a) Intact plantaris tendon seen in long axis medial to the ruptured Achilles tendon. (b) Intact plantaris tendon seen in short axis medial to the ruptured Achilles tendon. (c) Short‐axis imaging demonstrating a swollen and irritated sural nerve seen lateral to a ruptured Achilles tendon. FHL, Flexor Hallucis Longus muscle; LAT, lateral aspect of a short axis image; MED, medial aspect of a short axis image; SSV, small saphenous vein; T, tendon.

An intact plantaris tendon may be observed in short axis medially. An important consideration is that the sural nerve may become irritated by haematoma formation from an Achilles tendon rupture, or by tight application of a boot or plaster cast. When irritated, the sural nerve can appear swollen, hypoechoic, with an increased size in individual hypoechoic fascicles. A positive Sonographic Tinel's sign may be elicited when transducer pressure is applied over a swollen or irritated region of the sural nerve.^44^ Associated venous thrombosis can concurrently occur with an Achilles tendon rupture.^58^ Sonographic assessment of the veins of the gastrocnemius and soleus muscles as well as the deep posterior tibial and peroneal veins at a minimum should also be performed to exclude thrombosis^58^ (Figure 9).

## Dynamic sonographic imaging and measurement of the gap between ends of the acutely ruptured Achilles tendon

Dynamic sonographic imaging can allow direct observation of movement of acutely torn Achilles tendon ends when the foot is moved from plantar flexion to a neutral position. This can increase the confidence of diagnosing an acute complete rupture.^59^ Within 2 weeks following the acute injury, the tendon gap is usually observed to increase during dorsiflexion and decrease during plantar flexion. For example, a tendon gap of 12 mm with the ankle in neutral position may decrease to 5 mm with the ankle in maximum plantar flexion.^60^ The timing of the sonographic examination is important post‐injury. At 2–3 weeks post‐injury, if the patient has been immobilised, scar tissue formation will limit the movement of tendon ends.^56^ Hence, movement of ruptured tendon ends beyond 2–3 weeks post‐injury in an appropriately immobilised patient may not be sonographically observed with plantar flexion and extension. The deeper FHL muscle will, however, still be seen to move with plantar flexion. An Achilles tendon rupture is considered chronic at 4 weeks after the injury, and the tendon stumps may have retracted. At this stage, movement of tendon ends is not usually visible^9^ (Video S4).

**Figure 10:**
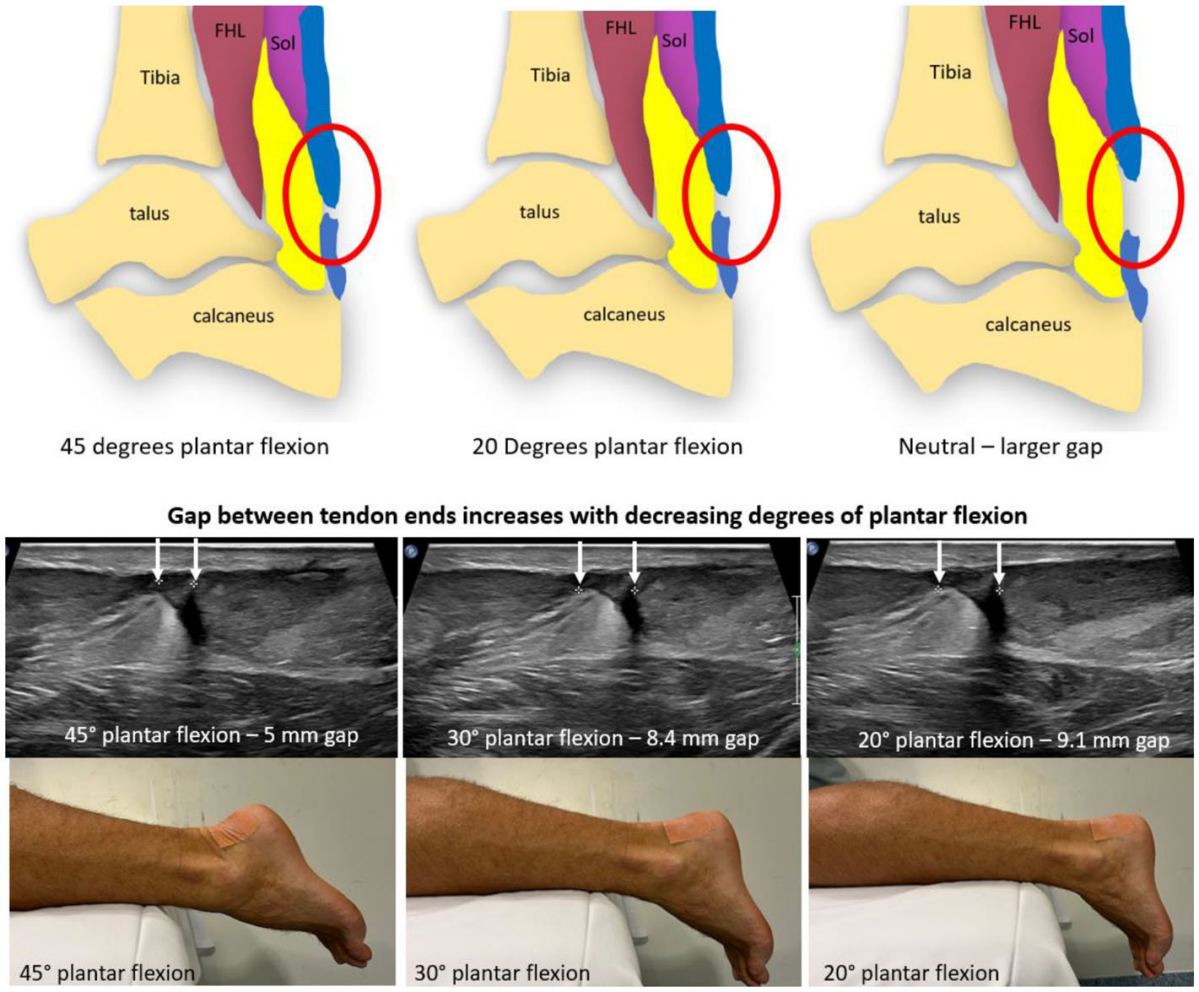
(a) Position of torn Achilles tendon ends (blue) with varying degrees of plantar flexion. As plantar flexion is reduced, the gap (outlined by red oval) between tendon ends becomes greater. (b) Long‐axis sonographic images acquired to measure the gap between ruptured Achilles tendon ends which increased with decreasing degrees of plantar flexion (demonstrated by accompanying photos). FHL, flexor hallucis longus muscle; Sol, soleus muscle.

When sonographically measuring the gap between ruptured tendon ends, the foot position and degree of plantar flexion must be documented. The foot can be passively moved to different degrees of plantar flexion ranging from maximum tolerated plantar flexion typically ranging from 30° to 45° to as close to neutral position as the patient can perform and/or tolerate. The tendon gap is usually measured in at least three different degrees of plantar flexion. If the patient reports discomfort with plantar extension, passive movement should be discontinued. Static images and measurements of the tendon gap can be acquired at any combination of 45°, 30°, 20°, and 10° plantar flexion and a neutral position. Neutral position is defined when the plantar aspect of the foot is perpendicular to the lower leg (Figure 10).

**Figure 11:**
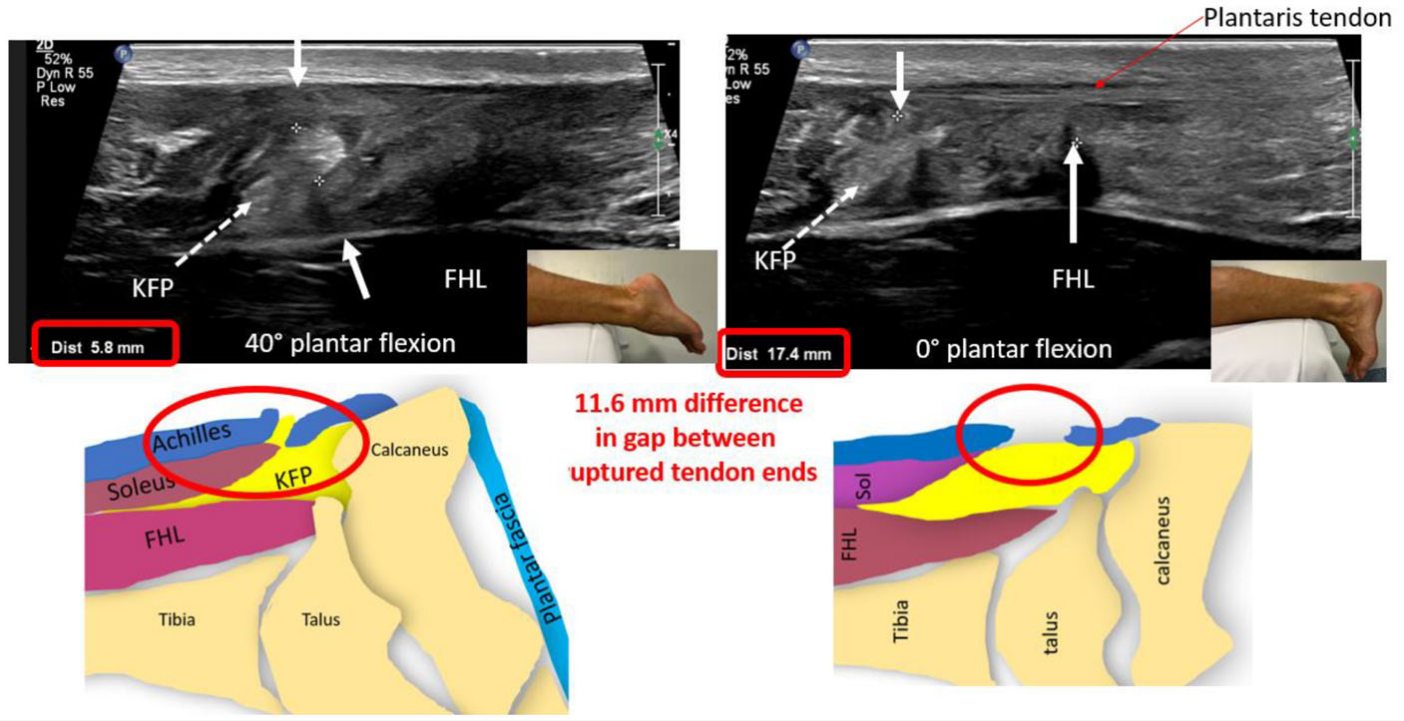
Long‐axis sonographic imaging of an acutely ruptured Achilles tendon, with associated anatomical schematics and photos of the ankle and foot position. The change in gap size when between 40° plantar flexion and 0° plantar flexion (neutral position) is demonstrated. When the ankle is in 40° plantar flexion, the tendon gap = 5.8 mm and when in neutral position, the tendon gap = 17.4 mm. The difference in the gap size is 11.6 mm. *Note*: the intact plantaris tendon can be seen in the medial aspect of the gap. The tendon ends nearly overlap with 40° plantar flexion with the proximal tendon end more superficially located, and the distal tendon end more deeply placed. FHL, flexor hallucis longus muscle; KFP, Kager's fat pad.

It is important to adapt the sonographic imaging gain when imaging and measuring the tendon gap, as posterior acoustic shadowing can occur at torn tendon ends. This can affect the accuracy of the calliper placement. Inaccurate calliper placement may result in under or overmeasurement of the tendon gap. Transducer rotation (clockwise or anticlockwise from the long‐axis position) may be required to identify the true tendon ends, as they may not align in the midline of the posterior ankle (Figure 11).

A tendon gap of 10 mm is often used as a minimum threshold value, beyond which operative management is considered.^10^, ^20^ A gap between tendon ends of >15 mm is considered to result in increased re‐rupture rates, hence surgical repair of these tendons is usually suggested.^6^, ^19^ There is a lack of consensus in the literature, as to what degree of plantar flexion should be used for these threshold values.^6^, ^20^ Consequently, further research is required to define the optimal gap measurement threshold for degrees of plantar flexion. Current protocols vary between institutions and departments and are often formulated after consulting relevant local stakeholders and reviewing the available literature.

At our institution, the sonographic gap measurements are acquired with the foot at maximum tolerated plantar flexion, and as close to neutral as tolerated, and a further degree of plantar flexion in between, with the angle of plantar flexion documented for each measurement. When the gap is 10 mm or less with the foot in maximum tolerated plantar flexion, conservative management is indicated. When the sonographically measured gap is between 10 and 15 mm with the foot at maximum tolerated plantar flexion, the patient is reviewed by the orthopaedic team, and shared decision‐making is undertaken incorporating physical and imaging findings, in addition to patient factors, to inform treatment. A tendon gap greater than 15 mm, measured with the foot at maximum tolerated plantar flexion, is used as an indicator for surgical repair. If overlapping of the tendon ends occurs with plantar flexion, the degree of plantar flexion where the ends overlap must be documented.

## Conclusion

Diagnostic imaging plays an important role in confirming a clinically suspicious acute Achilles tendon rupture. Ultrasound imaging remains the workhorse modality of choice, as it has an excellent ability to define the location and extent of an Achilles tendon tear, and presence of concurrent injuries. Sonographic reporting of the size of the diastasis in millimetres between ruptured Achilles tendon ends at different degrees of plantar flexion can be used to inform the decision regarding conservative or operative treatment. This is typically achieved with the foot at maximal tolerated plantar flexion which is patient specific. However, additional research is required to determine the optimal gap measurement thresholds to assist with deciding between operative vs. no‐operative management options. A gap of greater than 10 mm between ruptured ends of the Achilles tendon when the foot is in maximum tolerated plantar flexion requires orthopaedic review. Consideration of surgical treatment is often made taking into account patient factors and preferences.

## Authorship statement

The authorship listing conforms to the journal's authorship policy and all authors are in agreement with the content of the submitted manuscript.

## Funding

No funding information is provided.

## Conflict of interest

There are no conflicts of interest to declare.

## Supporting information


**Video S1.** Cine clip of short‐axis sonographic imaging of the uninjured Achilles tendon from superior to inferior.

**Video S2.** Cine clip demonstrating long‐axis sonographic imaging through an acutely ruptured Achilles tendon from lateral to medial aspects.

**Video S3.** Short‐axis sonographic imaging of acutely ruptured Achilles tendon from superior to inferior.

**Video S4.** Dynamic long‐axis sonographic imaging using plantar and dorsiflexion of the foot to demonstrate the change in size of the gap between acutely ruptured Achilles tendon ends.
